# FAMILY PERCEPTIONS OF THE EFFECTS OF COVID-19 ON OHIO LONG-TERM CARE FACILITIES

**DOI:** 10.1093/geroni/igae098.2996

**Published:** 2024-12-31

**Authors:** Oksana Dikhtyar, Jennifer Heston-Mullins

**Affiliations:** Miami University, Oxford, Ohio, United States; Miami University, Oxford, Ohio, United States

## Abstract

Family and friends are often the main source of emotional support and social contact for residents in long-term care facilities. The visitation restrictions imposed during the COVID-19 pandemic negatively impacted staff, family members, and residents, particularly those with cognitive impairment. However, there is limited literature on the impact that COVID-19 had on communication between residents and their family and friends, and communication between long-term care facilities and residents’ family and friends. To understand more about the effects of COVID-19, an online, state-wide survey was conducted which included questions regarding communication. A total of 1,686 involved family members or friends responded to the survey between September 2021 to May 2022. Phone calls were the most common method of communication between residents and their family and friends during visitation restrictions, followed by window visits and dropping off food and snacks. The frequency of communication also increased as a way for families to compensate for the lack of regular visits. Staff in most facilities used emails or postal mail, phone calls or text messages, and social media to communicate with family members. The amount of information respondents received from the facility affected their perceptions about the facility. These study findings can help improve facility-family communication and help facilities explore new ways of working with and engaging residents and families beyond the pandemic.

